# Cue relevance drives early quitting in visual search

**DOI:** 10.1186/s41235-024-00587-1

**Published:** 2024-08-26

**Authors:** Jeff Moher, Anna Delos Reyes, Trafton Drew

**Affiliations:** 1 Psychology Department, Connecticut College, 270 Mohegan Avenue, New London, CT 06320 USA; 2 University of Utah, Salt Lake City, USA; 3 Sirona Medical, San Francisco, USA

**Keywords:** Visual search, Cues, Early quitting, Salient distractors, Attentional capture, CAD

## Abstract

Irrelevant salient distractors can trigger early quitting in visual search, causing observers to miss targets they might otherwise find. Here, we asked whether task-relevant salient cues can produce a similar early quitting effect on the subset of trials where those cues fail to highlight the target. We presented participants with a difficult visual search task and used two cueing conditions. In the high-predictive condition, a salient cue in the form of a red circle highlighted the target most of the time a target was present. In the low-predictive condition, the cue was far less accurate and did not reliably predict the target (i.e., the cue was often a false positive). These were contrasted against a control condition in which no cues were presented. In the high-predictive condition, we found clear evidence of early quitting on trials where the cue was a false positive, as evidenced by both increased miss errors and shorter response times on target absent trials. No such effects were observed with low-predictive cues. Together, these results suggest that salient cues which are false positives can trigger early quitting, though perhaps only when the cues have a high-predictive value. These results have implications for real-world searches, such as medical image screening, where salient cues (referred to as computer-aided detection or CAD) may be used to highlight potentially relevant areas of images but are sometimes inaccurate.

**Supplementary Information:**

The online version contains supplementary material available at 10.1186/s41235-024-00587-1.

## Significance Statement

The present study examines how salient cues that sometimes highlight targets in visual search impact behavior. This is relevant for any type of visual search in which information from an initial reader is conveyed to a second human reader. The first reader may also be human, but in many cases artificial intelligence (AI) is used to make a first pass at identifying targets in a complex image. One example of this is in medical image screening, where computer-aided detection (CAD) signals are used to convey information from AI to a human observer. The negative effects that occur when these types of cues are false positives are not fully understood. In the present study, we find evidence to suggest that on the subset of trials in which cues are false positives, there are significant changes in strategy that cause participants to be more likely to miss targets that are present elsewhere. Moving forward, in any task in which information is conveyed from one searcher (human or not) to a second, human searcher, designers may want to consider alternative ways of highlighting information to avoid these early quitting effects. More research is needed to find out which alternative methods might be most effective.

**Supplementary Information:**

The online version contains supplementary material available at 10.1186/s41235-024-00587-1.

Conducting a visual search to find a target in a complex scene can be a difficult process. Anyone who has ever looked for a lost item on a messy desk can appreciate the challenge of completing a thorough, effective search. Part of that challenge is that humans lack a clear memory of where they have already looked (see, e.g., Le‐Hoa Võ & Wolfe, 2015 for a review) and that exhaustive search, in which we methodically check every item, is difficult if not impossible under most circumstances (e.g., Cousineau & Shiffrin, 2004; Schwark, et al., 2013; Wolfe et al., 2010). Decades of research have vastly improved our understanding of how the visual search process works (e.g., Chan & Hayward, 2013; Wolfe, 2021). Still, despite this knowledge and great levels of motivation in life-altering conditions like cancer screening, observers can fail to find targets. Medical image screening is one example—radiologists are experienced, well-trained, and highly motivated to find relevant information in medical images, and yet errors in image reading are consistently observed in experimental and clinical settings (e.g., Bruno et al., 2015; Rauschecker et al., 2020; Waite et al., 2020), leading to sometimes fatal outcomes for patients.

One factor that can impact the search process is that our attention does not always go where we would like. Perceptually salient objects that stand out from their surroundings due to their physical properties, for example, can capture our attention even when completely irrelevant to our current goals (e.g., Theeuwes, 1992). Historically, the impact of these salient distractors has largely been studied in contexts where a target is always present, and the impact of the distractor is measured as increased response times to find a target when a distractor is present compared to when it is absent.

In recent work, we explored the role of salient distractors in a visual search task in which targets were not always present (Moher, 2020). We found that when a salient distractor was present, and no target was present, participants spent less time searching the display before responding that the target was absent from the display. In addition, when a salient distractor was present, participants more frequently missed targets that were present. We concluded that the presence of a salient distractor caused participants to quit their search earlier than they otherwise would have, therefore gaining less information from the image and as a result being more likely to miss targets. Recent studies have expanded on the contexts in which this distractor-induced quitting effect may occur (e.g., Lawrence & Pratt, 2022; Lawrence et al., 2023; Lui et al., 2023; Wu & Pan, 2022).

One notable feature of Moher (2020) is that the distractor was entirely task-irrelevant. In the real world, however, salient signals are often relevant. Objects that stand out from their surroundings, such as a fire alarm, are meant to capture our attention. What remains uncertain is what the consequences are for the instances in which such a signal turns out to be a false alarm. That is, what happens in a visual search task if a salient signal sometimes cues a target, but sometimes cues a non-target? Does an inaccurate salient cue produce early quitting?

Drew et al. (2012) examined the impact of salient cues in a visual search task. Their experiment was inspired by the use of computer-aided detection (CAD), in which salient signals are used to convey information after artificial intelligence has marked a potentially relevant spot in a medical image. In their experiment, in the CAD condition, a red circle sometimes appeared. This circle sometimes highlighted a target, but it could also highlight non-targets, creating a false positive cue. The authors found that when the CAD mark highlighted a non-target, observers were more likely to miss a target that was present elsewhere in the display. They did not find differences in response time that are typically observed with early quitting, but this was not the focus of their experiment, and other differences (such as the presence of multiple CAD marks on some images) make interpretation of a distractor-induced quitting effect more difficult in their experiment. Nevertheless, the authors did find that when CAD marks were present, observers looked at less of the total display.

Some key gaps in knowledge about the distractor-induced quitting process remain. First, can something akin to distractor-induced quitting occur when a salient signal is sometimes relevant? Drew et al. (2012) provided evidence for an increase in miss errors when a sometimes salient signal turns out to be a false alarm. In addition, when multiple targets are present, a similar early quitting effect can occur in the form of subsequent search misses (e.g., Adamo et al., 2021), suggesting that bottom-up salience is not a necessary component of early quitting in visual search. Second, to what extent would an early quitting effect driven by task-relevant salient signals depend on the overall accuracy of the salient signal? For example, if every target was marked by a salient signal, there would be no need to search the rest of the display. Conversely, if the salient signal was never highlighting a target, it would be critical to search the rest of the display, though in Moher, 2020, the salient distractor was never the target and yet its presence triggered early quitting. Do observers adjust their search strategies as a function of the accuracy of the salient signal?

The answers to these questions are particularly relevant in the context of the growing number of real-world, high-stakes situations in which AI communicates with human observers, such as CAD. CAD provides a particularly interesting example, as there is debate to whether the implementation of CAD in clinical settings has improved outcomes or not (e.g., Fenton et al., 2011; Helbren et al., 2015). One potential reason that such a system would provide less of a benefit than might be anticipated is because of the downstream effects that occur on the instances in which the AI signal is incorrect. Indeed, in a series of papers, Kunar and colleagues have suggested that over-reliance on incorrect CAD marks may be a central reason for the underwhelming performance of CAD in practice (e.g., Kunar, 2022; Kunar & Watson, 2023; Kunar et al., 2017). Data from Moher (2020) and Drew et al. (2012) suggest one possible mechanism for why those false alarm trials could be particularly harmful in the form of early quitting, but it remains unclear whether early quitting can occur under these circumstances.

Although the costs of ineffective CAD systems are most clearly seen in applied settings such as diagnostic radiology, in order to understand the mechanisms that underlie behavior, it is advantageous to use artificial stimuli rather than real medical images. These stimuli provide the researchers with the ability to precisely manipulate factors such as target/distractor similarity, salience and prevalence. Importantly, as we have argued previously, although radiologists are amazingly adept at detecting cancer, they use the same ‘human search engine’ as naive subjects. It is therefore beneficial to use converging methods of both applied research with real stimuli and highly controlled stimuli to gain a more mechanistic understanding of findings observed in the field (Wolfe et al., 2016).

Therefore, in the present study, we explore this question by using salient cues to highlight items in a visual search task. We created an artificial CAD system that was designed to marginally improve performance on a difficult task. We have three conditions: a control condition with no cues, a high-predictive cue condition in which a cue circles the target 75% of the time when a target is present, and a low-predictive cue condition in which a cue circles a target only 25% of the time when a target is present. Our numbers were chosen to be broadly consistent with published reports of CAD accuracy. For instance, Birdwell et al. (2001) found that CAD correctly identified 73% masses that were initially missed. The low-predictive condition was set at 25% in order to generate a cue type that was sufficiently distinct as to provide a strong test of whether cue accuracy had any type of impact on performance. Given that we knew from pilot data that performance on this task without CAD-like cues was approximately a d’ of 2, we chose a 50% rate of cue presence on absent trials to ensure that the d’ of the artificial CAD system was useful in the high-predictive condition (a d’ higher than zero, but not better than the human alone).

Our two primary hypotheses of interest are as follows:

1. On target present trials, error rates will be higher when a cue highlights a non-target than when there is no cue present.
2. On target absent trials, RTs will be shorter when cues are present than when they are not present.

If a salient cue highlighting a non-target triggers early quitting, as salient signals have in our previous work, we would expect miss rates to be higher in this condition compared to a condition without a cue. Similarly, if participants are quitting early on these trials, RTs should be shorter when targets are absent compared to a condition where no cue is present. These two results would be consistent with early quitting, supporting the notion that task-relevant cues can trigger early quitting on the subset of trials when they do not highlight the target. If distractor-induced quitting occurs automatically, we might expect to observe similar effects in the high- and low-predictive cue conditions. Alternatively, if observers adapt to the overall accuracy of cues, we might expect more evidence of early quitting in the high-predictive condition, as the low-predictive cues provide less information about finding a target in the rest of the display. This would create a situation in which high-predictive cues produce the predicted results, but low-predictive cues produce data that look more similar to trials where cues are absent.

When the cue highlights a non-target and participants do successfully find a target elsewhere in the display, we would expect RTs to be longer than when no cue was present, consistent with the notion of attentional capture by the salient cue (e.g., Theeuwes, 1992). Finally, when cues highlight the target, we expect this to improve performance across both cue conditions significantly in the form of reduced miss rates and shorter RTs. Given that target discriminability is difficult, we expect that this benefit will be greater in the high-predictive condition where participants will prioritize the cue due to its higher reliability.

## Methods

The methods were preregistered at https://osf.io/cdxmu/. A total of 176 participants completed the experiment from the Connecticut College subject pool and on Prolific.co (100 male, 68 female, 4 non-binary, 4 not reporting; mean age: 38.1 years). Seven participants were from the subject pool at Connecticut College, and the remainder were collected online. Five participants were removed from analysis for d’ scores below 0 on the task. An additional 8 participants were removed for having more than 5 trials on which they eclipsed the 20-s timeout period. This latter exclusion criteria was not part of the original preregistration but was recommended by a reviewer expressing concerns about online participants not being engaged in the task. These cuts resulted in a total of 163 participants. Our a priori power analysis assuming a large effect size based on the key interaction in Moher (2020) estimated that we would achieve over 99% power with 57 participants in each of condition of the experiment.

Participants were required to have normal or corrected-to-normal color vision. Participants provided informed consent, were provided with monetary compensation or course credit, and the experimental protocol was approved by the Connecticut College Institutional Review Board. Prolific participants were required to reside in the USA, and have completed at least 100 studies with at least a 95% approval rate.

*Stimuli*. Custom software was created using Javascript and adapted sample scripts from PsiTurk to present stimuli (Gureckis et al., 2016). Images were generated ahead of time using custom Matlab scripts. Images of stimuli were placed behind a gray 1/f noise on a black background that was generated based on Castella et al., (2008) algorithm. This background noise is intended to emulate the scene statistics of breast tissue in mammograms.

The background was 600 by 600 pixels. Letter stimuli that appeared on top of the background were rotated offset “*Ts*” and “*L*”s that were gray and partly translucent. Twelve total items were present on each trial. On target present trials, one of these items was a rotated “*T*” and the remaining items were rotated “*L*”s. Rotations for each letter were randomly generated on a continuum up to 360˚. On target absent trials, all items were rotated “*L*”s. Each letter consisted of two intersecting lines, with each line created at approximately 50 × 10^1^ pixels. Each item was placed in a randomly selected location (without replacement) in a 4 × 4 grid measuring approximately 360 by 360 pixels and centered on the background, with grid locations equally spaced. Each item was then moved a randomly generated distance up to approximately 19 pixels in either direction along both the x and y axes. Opacity of the 1/f noise was set to 57% for practice trials and 61% for experimental trials.

On some trials, a red circle surrounded one of the items. These red circles are referred to in this manuscript as *cues,* since they sometimes surrounded the target. The cue had a radius of 43 pixels and was centered on the center of the item it was surrounding.

**Fig. 1:**
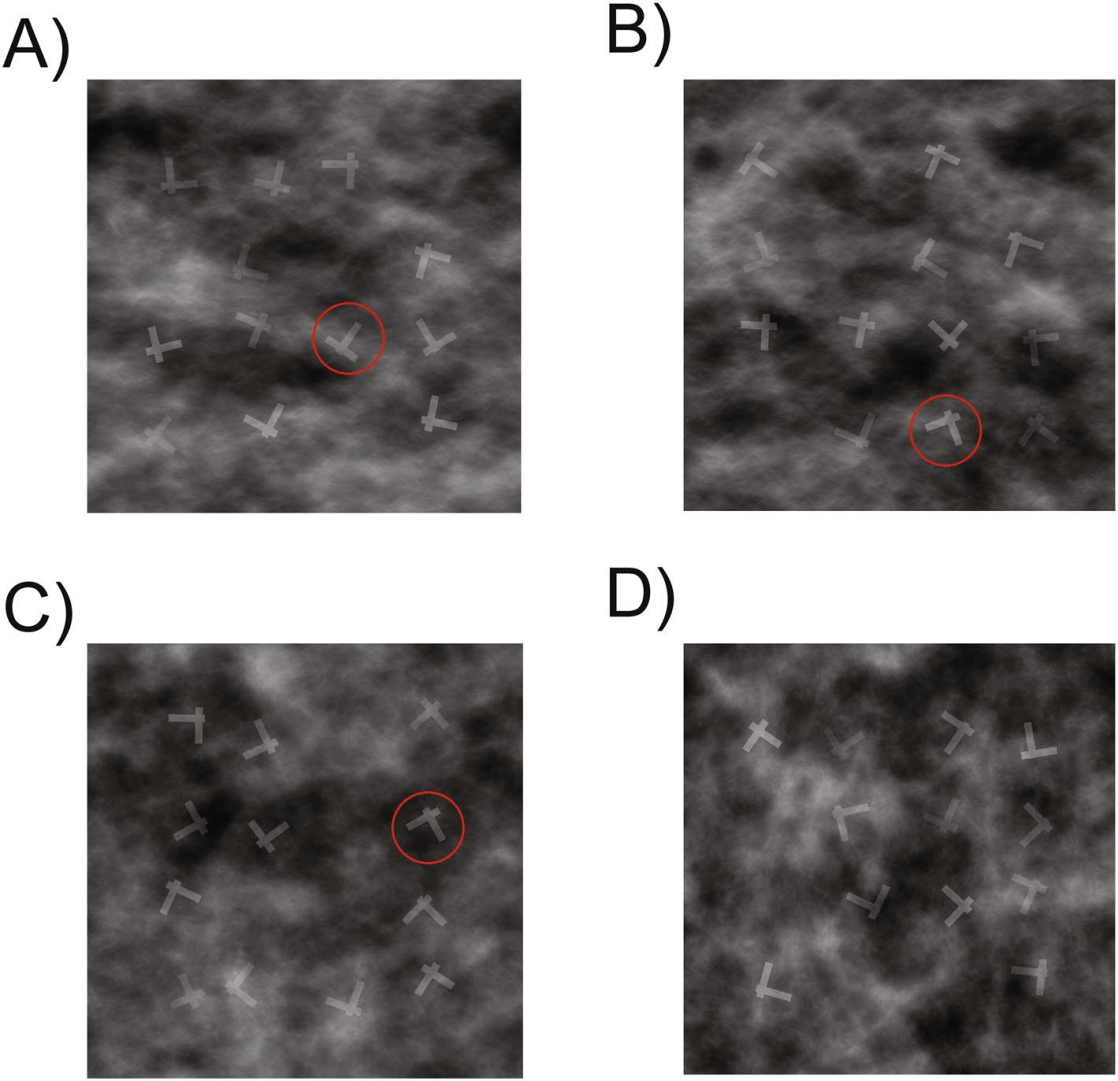
Example displays including **A** a cue on-target trial, **B** a cue off-target trial, **C** a cue off-target absent trial, and **D** a no-cue target absent trial

Targets were present on a randomly selected 1/3rd of all trials. There were three experimental conditions. In the control (no-cue) condition, there were no cues placed upon the screen on any trials. In the high-predictive cue condition, a red circle was sometimes presented surrounding one of the letters on the screen. On target present trials, cues were always present. The cue surrounded a target on a randomly selected 75% of target present trials, and a non-target on the remaining 25% of target present trials. On target absent trials, cues were present on a randomly selected 50% of trials, surrounding a non-target. The low-predictive condition was similar, except that on target present trials, cues surrounded the target on a randomly selected 25% of trials, while they surrounded a non-target on the remaining 75% of trials. Aside from cues, the images used across the three conditions were identical. Therefore, the only difference for a given trial across the conditions was the order in which it appeared in the experiment (randomly selected for each participant), and whether and where a cue appeared on that trial (see Fig. 1, for example, stimuli).

*Procedure.* Each participant was randomly assigned to either the no-cue, high-predictive, or low-predictive condition. Each trial began with a black fixation cross measuring 20 by 20 pixels presented at the center of the display for one second. Following this, the stimulus image was presented. Participants were instructed to press the “z” key if a rotated “T” target was present, and the “m” key if a target was absent. The stimuli remained onscreen until participants responded or until 20 s elapsed, at which point the trial was considered incorrect. These timeouts occurred an average of 1.15 times per participant, driven largely by a few participants who we suspect took a break from the task for a period of time. Written reminders of these key mappings were presented below the display throughout the experiment. At the end of each trial, there was a blank intertrial interval for one second.

Participants completed 50 practice trials with feedback in the form of the word “Correct!” or “incorrect” presented at the center of the display for one second following each response. This was followed by 2 blocks of 150 trials each, with a short break in between blocks. There was no feedback during this portion of the experiment. In the cue conditions, participants were instructed that cues would appear on some trials, sometimes circling the target but not always. Participants were not informed of the accuracy of cues, so the instructions for the two cue conditions were identical. At the end of the experiment, participants were asked to answer a question asking when a target was present, how likely it was that the red circle surrounded the target as opposed to a non-target. Participants were also asked to estimate the percentage of time that a target was present when a cue was present, and the percentage of time a target was present when a cue was not present. Participants used a slider from 0 to 100 percent to answer these three questions. Finally, participants were given an open-ended question to report anything else they noticed or thought about the cues (for the cue conditions), and about the experiment in general (for all conditions). This was done primarily to check for any concerns during data collection such as unclear instructions or errors, but was not analyzed further.

## Results

All statistical analyses below were pre-registered; pre-registration and data are available at https://osf.io/cdxmu/, along with raw data and data analysis code. For each variable of interest, a one-way between-subjects ANOVA was conducted to determine whether the condition (high-predictive, low-predictive, and control) impacted performance. Tukey’s pairwise comparisons were used to compare each individual condition in cases where the omnibus ANOVA was significant. Analyses were run in JASP (JASP Team, 2018). Planned contrasts to compare the two cue conditions to the control condition are reported in Table S1. Analysis of RTs only included accurate responses, and RTs were subjected to a recursive trimming procedure to remove outliers (Van Selst & Jolicoeur, 1994).

*Efficacy of cues.* To determine the impact of the presence of cues across conditions on overall performance and strategy, we examined dependent measures of d’, criterion, sensitivity (hit rate) and specificity (correct rejection rate).

There was a main effect of condition on d’, *F*(2,160) = 7.89, *p* < 0.001, *η*_*p*_^2^ = 0.09. D’ was higher in the high-predictive condition (2.87) compared to the control condition (2.12), *p* < 0.001. D’ in the low-predictive condition was 2.53—this did not differ significantly from the high-predictive (*p* = 0.08) or control conditions (*p* = 0.19). There was also a main effect of criterion, *F*(2,160) = 3.93, *p* = 0.02, *η*_*p*_^2^ = 0.05. Participants were less conservative in the high-predictive condition (0.59) compared to the low-predictive condition (0.77), *p* = 0.02. There was no difference between the control (0.71) and either other condition, *p*s > 0.17.

There was a main effect of condition on hit rate as well, *F*(2,160) = 12.98, *p* < 0.001, *η*_*p*_^2^ = 0.14. The hit rate in the high-predictive condition (78%) was significantly higher than the low-predictive condition (67%) or the control condition (63%), *p*s < 0.01. The latter two did not differ statistically, *p* = 0.27. There was no main effect of condition on correct rejection rate, *F*(2,160) = 2.05, *p* = 0.13, *η*_*p*_^2^ = 0.03.

Together, these data suggest that the presence of cues improved performance when the cues were highly predictive of targets, but not so when the cues were less accurate. This was mostly reflected in an increase in hit rate in the high-predictive condition, with no concurrent cost in correct rejection rate. In other words, the presence of highly predictive cues did improve participants’ ability to find targets, as expected.

*Accuracy.* To determine the impact of cues on performance at a more fine-grained level, we first focused on target present trials. We conducted a one-way ANOVA across the three experimental conditions, for trials in which a target was present and accurate cues were included for the relevant cue conditions. We included all target present trials for the control condition in this analysis. For completeness, in Table S2, we report the outcome of paired samples t-tests for comparisons of each cue condition against only its exact matched control images from the control condition (rather than all target present trials from the control condition). A similar approach was used for trials where the cue did not mark the target. The outcome of these tests was broadly consistent with the outcome of the tests that included all target present trials from the control condition reported here.

For trials where the cue marked the target, there was a main effect of condition, *F*(2,160) = 49.18, *p* < 0.001, *η*_*p*_^2^ = 0.38. Accuracy in the high-predictive condition (89%) did not statistically differ from the low-predictive condition (84%), *p* = 0.16. Both of these conditions showed greater performance than the control condition (63%), *p*s < 0.001. These data are not surprising, demonstrating that the presence of a cue surrounding a target increases the accurate detection of that target.

**Fig. 2:**
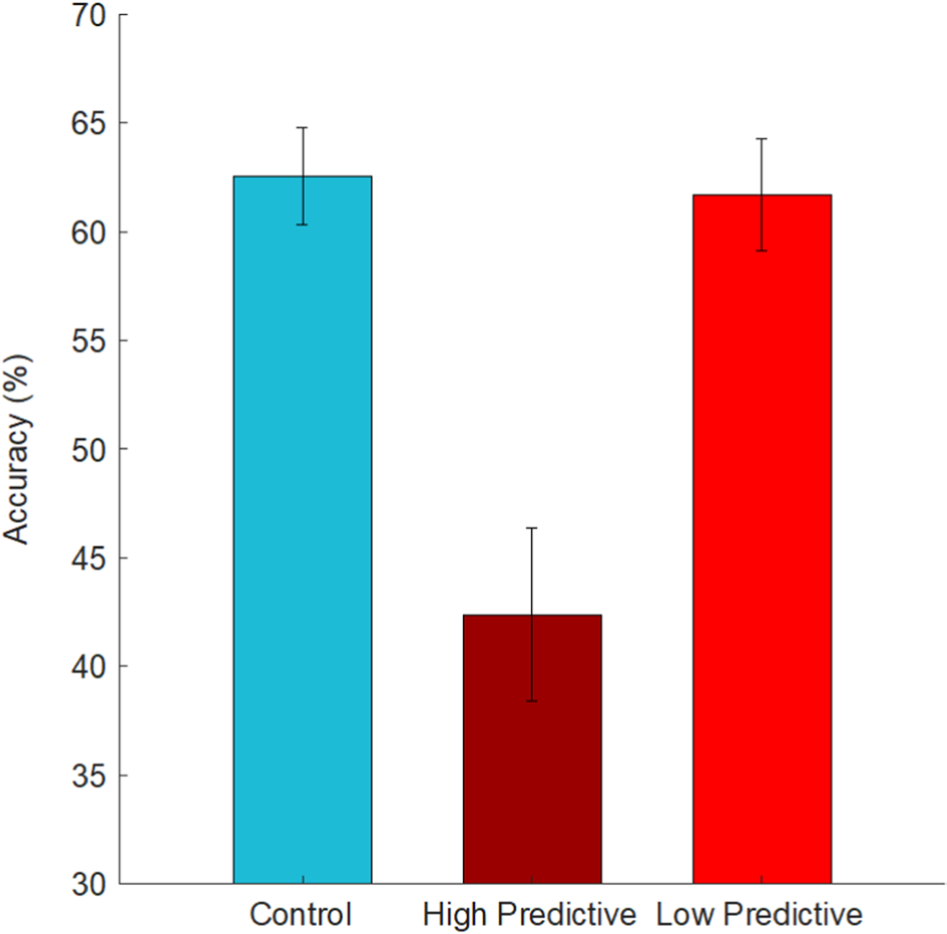
Target present response accuracy as a function of experimental condition on trials in which the cue was not surrounding the target. Error bars represent standard error of the mean

Our main analysis of interest indicated in primary hypothesis #1 was in assessing accuracy when a cue appeared on a target present trial, but did not mark the target. For these trials, there was a main effect of condition, *F*(2,160) = 14.31, *p* < 0.001, *η*_*p*_^2^ = 0.15. This demonstrates that cues once again had an impact. However, this time the effect was in the opposite direction. In the high-predictive condition, accuracy was significantly lower (42%) than in the low-predictive (62%) or control (63%) conditions, *p*s < 0.001 (Fig. 2). There was no difference between the low-predictive and control conditions, *p* = 0.98.

These data suggest that despite an overall benefit to the presence of cues, there is also a cost. When cues are generally reliable, on the subset of trials for which they miss the mark, they decrease accuracy significantly. This is in line with the findings of Drew et al. (2012) and predictions from distractor-induced quitting (Moher, 2020). However, in the current study, this only occurs when the cues are highly predictive.

An important note is that in the cue conditions, trials in which no cue appeared were always target absent trials. We made this design decision in order to maximize the relevance of the cue while also providing a within-subjects comparison for the presence of cues on target absent trials. Still, it is possible that participants recognized this pattern and adjusted their behavior accordingly, recognizing that the absence of a cue indicated that no target was present. Therefore, we compared target absent trials with no cue present across the three conditions. If participants were aware of the general contingency described above, we would expect accuracy to be higher in the cue conditions for these trials. However, we found no effect of condition on accuracy for cue absent trials, *F*(2,160) = 1.48, *p* = 0.23, *η*_*p*_^2^ = 0.02.

To gain more power for this comparison, we also conducted within-subjects comparisons of responses on target-absent trials as a function of whether a cue was present or absent (within-subjects) and whether it was the high- or low-predictive condition (between-subjects). There was no main effect of cue presence or condition on accuracy, *p*s > 0.82. There was a significant interaction, *F*(1,105) = 4.57, *p* = 0.04, *η*_*p*_^2^ = 0.04. Numerically, cues decreased the false alarm rate in the high-predictive condition from 6 to 4%, and increased the false alarm rate in the low-predictive condition from 5 to 6%. However, neither change reached statistical significance, *p*s > 0.09, making this interaction difficult to interpret.

*Response time.* We conducted the same set of analyses on response times (RTs). On trials in which the cue surrounded the target, there was a main effect of condition, *F*(2,160) = 57.48, *p* < 0.001, *η*_*p*_^2^ = 0.42. RTs were shortest in the high-predictive condition (1547 ms), followed by the low-predictive condition (2414 ms) and the control condition (3266 ms). Comparisons among all conditions were significant, *p* < 0.001. Thus, participants appeared to prioritize searching the item surrounded by the cue relatively quickly, and this prioritization was graded according to the overall predictive accuracy of the cue.

There was no effect of condition on target present trials in which a cue surrounded a non-target, *F*(2,160) = 1.01, *p* = 0.37, *η*_*p*_^2^ = 0.01. This was somewhat surprising—we expected that when a cue surrounded a non-target, something akin to an attentional capture effect would occur, because participants would be initiating their search with a guaranteed non-target. Instead, RTs were not significantly different on these trials as a function of cue presence. This result is covered in more detail in the general discussion.

The most critical condition of interest for RTs was related to our hypothesis #2. We assessed whether cues that surrounded a non-target might trigger early quitting on the subset of trials on which no target was present. If the presence of a cue on these trials produces shorter RTs in one or both of the cue conditions, it would suggest that the incorrect cues trigger early quitting. On these trials, there was a significant effect of condition, *F*(2,160) = 11.34, *p* < 0.001, *η*_*p*_^2^ = 0.12. RTs were considerably shorter in the high-predictive condition (3282 ms) compared to both the low-predictive (4511 ms) and control (4448 ms) conditions, *p*s < 0.001. The difference between the low-predictive and control conditions was not significant, *p* = 0.98. Thus, in the high-predictive condition, we indeed saw evidence of early quitting generated by the presence of cues.

On target absent trials when no cue was present, there was also an effect of condition, *F*(2,160) = 6.45, *p* = 0.002, *η*_*p*_^2^ = 0.08. Similar to the target absent condition with cues, RTs were notably shorter in the high-predictive condition (3534 ms) compared to the low-predictive (4427 ms) and control (4379 ms) conditions, *p*s < 0.01. The low-predictive and control conditions did not differ from each other, *p* = 0.98.

**Fig. 3:**
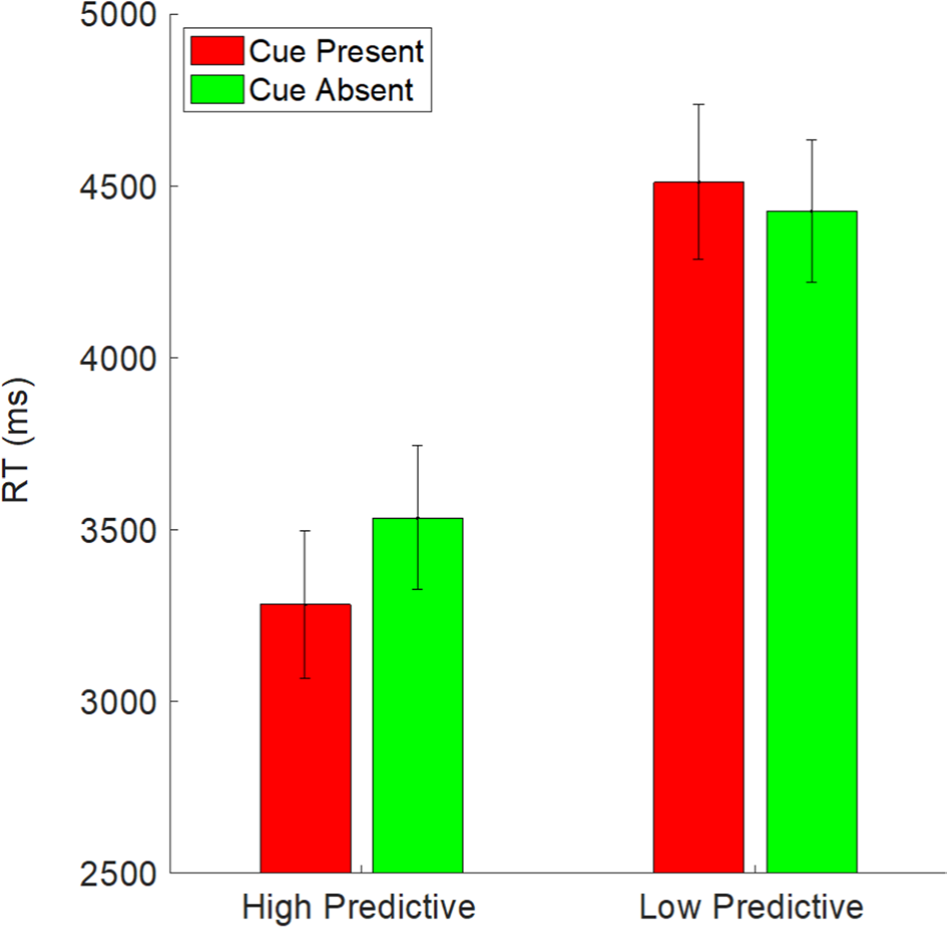
Target absent response time data as a function of whether a cue was present or absent in the two cueing conditions. Error bars represent standard error of the mean

This result makes the interpretation of early quitting a bit more difficult, as it appears that participants in the high-predictive condition adopted a strategy of quitting early more broadly, rather than just when a cue appeared. However, as with accuracy, we are also able to get a better picture through a within-subjects comparison of what happens when cues are present or absent within both cue conditions. Here, there was no effect of cue, *F*(1,105) = 2.79, *p* = 0.1, *η*_*p*_^2^ = 0.03, and a main effect of condition, *F*(1,105) = 12.62, *p* < 0.001, *η*_*p*_^2^ = 0.11, along with a significant interaction, *F*(1,105) = 11.12, *p* = 0.001, *η*_*p*_^2^ = 0.1. Simple main effects revealed that there was no effect of cues in the low-predictive condition, *p* = 0.23. However, in the high-predictive condition, RTs were significantly shorter when a cue was present (3282 ms) compared to when it was absent (3534 ms), *p* = 0.001. Figure 3 clearly illustrates both a general speeding of RTs in the high-predictive condition relative to the low-predictive condition on target absent trials, along with an effect specific to the presence of cues in the high (but not low) predictive condition. The latter effect is consistent with early quitting—when a cue surrounds a non-target, participants terminate their search earlier than they otherwise would have.

Together with the accuracy data, these results demonstrate that high-predictive cues generate early quitting when the cue highlights a non-target. This suggests a specific mechanism by which cues that sometimes highlight a target can have a negative effect when they are inaccurate, even if they improve overall performance. Furthermore, we failed to find a similar effect with more inaccurate cues. Thus, there is at least some strategic component to this early quitting effect with task-relevant cues.

*Self-report.* At the end of the experiment, we asked participants in the cue conditions to indicate the likelihood that a cue, if present, highlighted a target. Because this calculation also includes target absent trials, the correct answer would be 37.5% of the time in the high-predictive condition, and 12.5% of the time in the low-predictive condition. Participants’ actual answers were 31.8% in the high-predictive condition and 23.2% in the low -predictive condition, *t*(103) = 2.13, *p* = 0.04, *cohen’s d* = 0.42.

We also asked participants to estimate the probability that a target was present elsewhere in the display when the cue marked a non-target item. Here, the true probabilities are flipped from the above, with the correct answer being 37.5% in the low-predictive condition and 12.5% in the high-predictive condition. The answers participants gave were 36.8% in the low-predictive condition and 23.0% in the high-predictive condition, *t*(103) = 3.03, *p* = 0.003, *cohen’s d* = 0.59. Therefore participants did exhibit some explicit awareness of the difference in predictiveness of the cues between the two conditions, ruling out the possibility that the effects observed in RT and accuracy were driven by completely implicit processes, though participants underestimated the accuracy of the high-predictive cues and overestimated the accuracy of the low-predictive cues.

Finally, we asked participants to estimate how frequently a target appeared when no cue was present. The true answer here was 0%—we included this condition in order to generate RT comparisons on target absent trials as a function of cue presence, but wanted to maximize the number of cue off-target trials since that was a key condition of interest. Therefore, there was a macro signal in the sense that participants might realize that targets were never present when no cue was present. If they did realize this, we would expect RTs to be significantly shorter in the cue conditions on the cue absent trials, but this was not observed. Furthermore, we would expect participants’ answers here to be near 0%. Instead, the mean estimate from our participants was 26.6% in the low-predictive condition and 17.8% in the high-predictive condition, *t*(102) = 2.94, *p* = 0.02, *cohen’s d* = 0.48. Both of these groups’ estimates were significantly higher than zero, *p*s < 0.001.

## Discussion

The main goal of the current study was to determine whether early quitting occurred with task-relevant cues when those cues highlighted a non-target. We proposed two hypotheses to test this possibility. First, we hypothesized that accuracy would be lower on trials where a cue highlighted a non-target compared to a control condition with the exact same search image but no cue present (Hypothesis #1). Second, we hypothesized that RTs on target absent trials would be shorter when cues were present than when cues were absent (Hypothesis #2). Both of these hypotheses were supported in the high-predictive condition. Thus, we conclude that when the overall accuracy of cues is relatively high, these cues do trigger early quitting on the subset of trials in which they highlight a non-target.

The cause of this early quitting may be bottom-up, as we suggested in Moher (2020), or may be a top-down over-reliance on cues (e.g., Kunar et al., 2017) as participants learn that cues in the high-predictive condition usually do highlight targets when targets are present. Alternatively, it is possible that a combination of these factors is influencing behavior. In the low-predictive condition our two hypotheses were not supported. This supports a top-down, strategic element to early quitting with task-relevant cues, such that they only appear to trigger early quitting when they are generally accurate. However, this is not because participants were not using the cues in the low-predictive condition. Both RT and accuracy data demonstrate that participants were prioritizing searching the cued item even in the low-predictive condition. So why did these cues not trigger early quitting? One possibility is simply that it was rational to expect a target to be more likely to be present when the cue was incorrect in the low-predictive condition compared to the high-predictive condition. That is, in the low predictive condition, it was indeed more likely that a target was present elsewhere in the display if the cue surrounded a non-target. Note, however, that this is still a practical problem in situations like CAD. Furthermore, if participants are capable of searching the rest of the display more thoroughly when cues are not accurate, as they exhibited in the low-predictive condition, there is little cost to doing so in the high-predictive condition other than time. One critical area for future research is to determine the extent to which these effects occur even when the stakes are higher, and how quickly participants learn and adapt based on cue accuracy.^2^

A second factor that may have influenced behavior in the current study is the difficulty of the task. Even in the high-predictive condition, cues highlighting the target only produced accuracy at 89%. This may have been partly due to motor errors where participants recognize they made a mistake only after responding (e.g., Fleck & Mitroff, 2007), or may be attributable to noise due to online data collection.^3^ Furthermore, the difficulty of discriminating a target “*T*” based on its offset from center may have made targets on some trials very difficult to identify. In future research, task difficulty may be another factor to explore that interacts with the predictiveness of the cue in affecting behavior.

It is also interesting that early quitting was not observed in the low-predictive condition but was observed in Moher (2020), where the salient signal was never the target. Why the discrepancy in these findings? One key difference is that in Moher (2020), there was no reason to start the search with the salient item. If attention was captured by that item, it was presumably involuntary. Whereas, in the present study, it is not a bad strategy even in the low-predictive condition to start by attending the cued item. The extent to which early quitting in the two sets of experiments is related is not entirely clear. As mentioned above, there may be combined bottom-up and top-down effects of the cue, which was perceptually salient and task-relevant, in the current study. Still, the results of the present study demonstrate that early quitting triggered by task-relevant salient cues differs from early quitting triggered by task-irrelevant salient distractors, in that it appears to depend on the informational value of the cue. One avenue for future research is to determine whether incentives or explicit instructions can change behavior in the high-predictive condition (e.g., Cox et al., 2021), though in other domains it has proven difficult to adjust quitting threshold in these ways (e.g., Wolfe et al., 2007). In one recent example exploring instructions, Kunar and Watson (2023) found that explicit instructions could alter the negative influences of CAD-like cues in visual search. When participants were instructed to ignore cues entirely, miss rates and false alarms for inaccurate cues were reduced. Interestingly, in this condition, there was still evidence of over-reliance on cues, though it was reduced compared to other instruction conditions. These results further support the notion that both top-down and bottom-up effects drive behavior in search tasks involving salient cues.

Unlike in Moher (2020), there was no evidence of an RT cost to inaccurate cues on target present trials. In Moher (2020), we took this as evidence of attentional capture from the task-irrelevant distractor. One possible explanation for not observing a similar effect in the current study is that the cues may have initiated the onset of search earlier relative to the control conditions. Thus, while the first item searched may have been a non-target when the cue was inaccurate, the search itself may have started sooner, offsetting some of this cost. Future eyetracking studies will be able to test this possibility directly. Numerically, when the cue was highlighting a non-target, RTs were longer in the high-predictive condition (3389 ms) and the low-predictive condition (3583 ms) compared to the control condition (3266 ms), so it is also possible that the study was underpowered to detect a relatively small effect similar to attentional capture. However, given that we observed an effect size of *η*_*p*_^2^ = 0.01 in our sample, this effect would likely be quite small indeed if it did exist at all in a larger sample. A second important distinction is that the red, unfilled circle in the current design may not have been as strong of a salient signal as the red bars used in Moher (2020).^4^ In future studies, varying the salience of the signal might help distinguish the role that visual salience itself, rather than salience combined with task relevance, plays in these types of search tasks.

In a related study, Russell and Kunar (2012) used a similar approach to studying the effects of salient cues in visual search. However, in their study, RTs were shorter when no cue appeared compared to when a cue highlighted a non-target. One possible explanation for the discrepancy between those results and the present study is that in Russell and Kunar (2012), most of the data came from a low-prevalence condition in which targets only appeared on 2% of all trials. In that condition, if a cue was present and not highlighting a target, the probability that it was a target absent trial was extremely high. Although those authors found similar RT results in a high-prevalence data set, the high-prevalence data were collected in a much shorter session that was usually completed after a significant number of low-prevalence condition trials had already been completed. Thus, when completing high-prevalence trials, participants had likely learned conditional probabilities from the low prevalence condition which may have impacted performance, consistent with prior work showing that bias shifts associated with prevalence can lag behind actual changes in prevalence (e.g., Wolfe & Van Wert, 2010). Taken together with our data, this suggests the possibility that learning of conditional cue probabilities is an important factor in determining how cues impact behavior in visual search, and future research will be necessary to better understand the nature of this learning process.

The present data provide novel insight into the ways in which initial shifts of attention toward cued or salient items might alter subsequent strategies. Early quitting has been observed previously as a function of the number of targets present (e.g., Berbaum et al., 2015), or the global or local target prevalence (e.g., Peltier & Becker, 2016; Wolfe & Van Wert, 2010). Our recent work suggested that the mere presence of salient distractors could also trigger early quitting (Moher, 2020). Here, we find that inaccurate cues can serve as another trigger of early quitting in visual search. This change in quitting strategy appears to occur in part after the trial has already begun, rather than based on overall probabilities, because whether a cue is present is not known prior to the trial onset. However, we also observed some more general change in quitting strategy, as the RTs on target absent, cue absent trials were quite different between the high- and low-predictive conditions. Thus, there are likely multiple mechanisms involved in adjusting search strategies in the present study—a global change as a function of how often the cue is accurate, and a local change as a function of whether a cue is present.

The current data also have implications for applied visual search settings, such as airport baggage X-rays or medical image screening. There is growing interest in using AI to help overburdened radiologists. This has led to a rapid increase in the number of AI-enabled devices that have been approved by the FDA. In fact, 87% of the AI-enabled medical devices that received 510(k) clearance (meaning that they have been approved as safe and effective by the Federal Drug Administration) in 2022 focus on radiology (https://www.fda.gov/). Many of these devices aim to improve radiologist performance by providing information to the clinician, often by using salient signals to indicate the location of a possible lesion in the form of CAD marks. However, recent work from Kunar and colleagues has suggested that an important predictor of adverse outcomes associated with CAD is an over-reliance on CAD marks (e.g., Kunar, 2022; Kunar & Watson, 2023; Kunar et al., 2017). Their work, as well as previous work from Drew et al. (2012), suggests that these effects are particularly strong when the cue (the CAD mark) is presented at onset rather than being presented as ‘second-reader’ where it is deployed after the clinician has made an initial decision. The current results, along with this previous literature, suggest that designers of such systems should be especially cautious about how cues are presented and what happens when those cues are inaccurate. It is even possible that the effects observed here could be further exacerbated in real-life situations where the accuracy of the cue is not independent from the difficulty of the search for a given image. In other words, cues might be more likely to be inaccurate on images where it is more difficult for a human observer to find a target. It also remains to be seen how cues interact with target prevalence, as targets are often rare in medical image searches, and target prevalence can drastically impact search performance (e.g., Wolfe et al., 2007). It is noteworthy that early quitting effects were observed at lower target prevalence in Moher (2020), and Russell and Kunar (2012) found similar effects of cues in visual search at both high- and very low-prevalence levels, so it may be the case that what we observed in the present study would still occur with much lower target prevalence, but future research is needed to confirm this possibility.

With constantly evolving AI image processing capabilities, it is likely that communication between AI and humans will become more prominent in the coming years across more domains. Therefore, it is important that we better understand exactly how humans respond when given potentially inaccurate information in a visual search task. The present data suggest at least one context in which the downstream effects of visual cues can harm search performance on a subset of trials. Of course, overall accuracy was higher in the high-predictive condition than in the other conditions, so we do not claim that such cues are invaluable as a whole. Rather we argue that understanding the specific ways cues interact with human observers is critical for optimizing performance and minimizing harmful errors.

## Conclusions

The present results suggest that when a cue is largely reliable during visual search, its presence can trigger early quitting when it is a false positive. However, less reliable cues do not produce the same pattern of results. This finding has implications both for understanding basic visual search and for applied settings where cues may be used to aid searchers in finding targets in complex images, such as medical image screening. For any system that uses AI to communicate information about a visual image to a human observer, our findings highlight potential hidden costs that should be addressed.

### Supplementary Information

**Additional file 1.**

## Data Availability

The datasets generated and/or analyzed during the current study are available in the open science framework repository, https://osf.io/cdxmu/. The experiment reported here was also preregistered at https://osf.io/cdxmu/

## Abbreviations

AI: Artificial intelligence
CAD: Computer-aided detection
RT: Response time

## Footnotes

1 Images were initially created at 800 × 800 pixels and scaled down to 600 × 600, so these pixel values are approximated based on scale adjustment.

2 We thank Hayward Godwin for this suggestion. As an exploratory analysis, we compared accuracy on high-predictive trials vs. control trials for a few key conditions, looking only at the first 10% of the experiment (the first 30 trials). Among these trials, we found that when the cue highlighted a non-target, accuracy in the high-predictive cue trial was lower (48%) than control trials (65%). We also found that when a cue was present on target absent trials, RTs were much shorter in the high-predictive cue trials (3432 ms) than the control trials (4920 ms). Despite the small amount of trials, both these differences were significant, ps < .01. This suggests that learning occurs quite quickly in this task, but again this is only an exploratory analysis that may be instructive for future research.

3 We thank Melina Kunar for raising these possibilities

4 We thank an anonymous reviewer for raising this possibility
