# Supplementary material for: Cue relevance drives early quitting in visual search
Source: Cogn Res Princ Implic. 2024 Aug 26;9:54. doi: 10.1186/s41235-024-00587-1 (PMC11345343; doi:10.1186/s41235-024-00587-1)
Supplement: Supplementary file 1 — Additional file 1. [file 41235_2024_587_MOESM1_ESM.docx]

**Supplementary Materials**

In our preregistration, we committed to conducting planned contrasts that weighted the two CAD conditions against the control condition. Because there were a number of measures for which the two CAD conditions differed, the individual Tukey contrasts (which were also preregistered) provided a more accurate parsing of the data. For completeness, we report the results of all the planned contrasts for each analysis in the table below.

In each condition for which the matched control images were different for the two CAD conditions, we conducted separate paired-samples t-tests to compare the relevant CAD condition to only the exact matched subset of images from the control condition. We report the table of these outcomes below – none of them change our interpretation of the primary results reported in the manuscript.
